# The impact of menopausal hormone therapy (MHT) on cardiac structure and function: Insights from the UK Biobank imaging enhancement study

**DOI:** 10.1371/journal.pone.0194015

**Published:** 2018-03-08

**Authors:** Mihir M. Sanghvi, Nay Aung, Jackie A. Cooper, José Miguel Paiva, Aaron M. Lee, Filip Zemrak, Kenneth Fung, Ross J. Thomson, Elena Lukaschuk, Valentina Carapella, Young Jin Kim, Nicholas C. Harvey, Stefan K. Piechnik, Stefan Neubauer, Steffen E. Petersen

**Affiliations:** 1 William Harvey Research Institute, NIHR Biomedical Research Centre at Barts, Queen Mary University of London, Charterhouse Square, London, United Kingdom; 2 Division of Cardiovascular Medicine, Radcliffe Department of Medicine, University of Oxford, Oxford, United Kingdom; 3 Department of Radiology, Severance Hospital, Yonsei University College of Medicine, Seoul, South Korea; 4 MRC Lifecourse Epidemiology Unit, University of Southampton, Southampton General Hospital, Southampton, United Kingdom; 5 NIHR Southampton Biomedical Research Centre, University of Southampton and University Hospital Southampton NHS Foundation Trust, Southampton, United Kingdom; Universitair Medisch Centrum Utrecht, NETHERLANDS

## Abstract

**Background:**

The effect of menopausal hormone therapy (MHT)–previously known as hormone replacement therapy–on cardiovascular health remains unclear and controversial. This cross-sectional study examined the impact of MHT on left ventricular (LV) and left atrial (LA) structure and function, alterations in which are markers of subclinical cardiovascular disease, in a population-based cohort.

**Methods:**

Post-menopausal women who had never used MHT and those who had used MHT ≥3 years participating in the UK Biobank who had undergone cardiovascular magnetic resonance (CMR) imaging and free of known cardiovascular disease were included. Multivariable linear regression was performed to examine the relationship between cardiac parameters and MHT use ≥3 years. To explore whether MHT use on each of the cardiac outcomes differed by age, multivariable regression models were constructed with a cross-product of age and MHT fitted as an interaction term.

**Results:**

Of 1604 post-menopausal women, 513 (32%) had used MHT ≥3 years. In the MHT cohort, median age at menopause was 50 (IQR: 45–52) and median duration of MHT was 8 years. In the non-MHT cohort, median age at menopause was 51 (IQR: 48–53). MHT use was associated with significantly lower LV end-diastolic volume (122.8 ml vs 119.8 ml, effect size = -2.4%, 95% CI: -4.2% to -0.5%; p = 0.013) and LA maximal volume (60.2 ml vs 57.5 ml, effect size = -4.5%, 95% CI: -7.8% to -1.0%; p = 0.012). There was no significant difference in LV mass. MHT use significantly modified the effect between age and CMR parameters; MHT users had greater decrements in LV end-diastolic volume, LV end-systolic volume and LA maximal volume with advancing age.

**Conclusions:**

MHT use was not associated with adverse, subclinical changes in cardiac structure and function. Indeed, significantly smaller LV and LA chamber volumes were observed which have been linked to favourable cardiovascular outcomes. These findings represent a novel approach to examining MHT’s effect on the cardiovascular system.

## Introduction

The effect of menopausal hormone therapy (MHT), previously known as hormone replacement therapy, on cardiovascular health in post-menopausal women remains controversial and unclear. Extensive observational data had suggested MHT to be cardioprotective [1–3], leading to MHT being routinely prescribed for both primary and secondary prevention of coronary heart disease (CHD). However, subsequent data from the Women’s Health Initiative (WHI) and Heart and Estrogen/Progestin Replacement Study (HERS) studies cast doubt on the beneficial cardiovascular effects of MHT [4–6]; this was reflected in learned societies’ clinical guidance concerning MHT’s role in CHD prevention [7,8]. The most recent randomised trial data on the subject arose from the Danish Osteoporosis Prevention Study [9], which indicated that women taking MHT had a reduced risk of the composite endpoint of mortality, heart failure and myocardial infarction but the study has been subject to criticism [10]. In more recent work, again from the WHI, there was no difference in cardiovascular mortality in MHT users compared to placebo, although the authors themselves state that cause-specific mortality data should be interpreted “cautiously” [11]. it has been suggested that commencement of MHT in the perimenopausal transition or early menopause is not associated with increased risk of CHD compared to when treatment is administered at a later stage. This is known as the “timing hypothesis” [12,13].

The UK Biobank is an ongoing, large-scale, population-based study designed to examine determinants of health in middle and old age [14]. Besides extensive collection of health questionnaire data, biological samples and physical measurements, it has incorporated cardiovascular magnetic resonance (CMR) imaging–the gold standard for analysis of cardiac structure and function–to provide detailed imaging phenotypes [15]. At present, there is a paucity of data on the effects of MHT on left ventricular (LV) and left atrial (LA) volumes and function, alterations in which are markers of subclinical cardiovascular disease and have prognostic implications.

This cross-sectional study aims to examine the impact of MHT on left ventricular and left atrial structure and function, as assessed by CMR, in a large, population-based cohort.

## Methods

### Study population

The UK Biobank is a versatile scientific resource, in which questionnaire data, physical measurements and biological samples were collected from over 500,000 individuals aged 40–69 between 2006 and 2010 registered with the UK National Health Service; the study protocol has been described in detail previously [14]. Additionally, the UK Biobank imaging enhancement study is ongoing with the aim of performing, in a single visit, brain, heart, whole body, carotid artery, bone and joint imaging in 100,000 of the original 500,000 participants. Cardiovascular magnetic resonance imaging (CMR) was selected as the modality of choice for heart imaging. The study population presented here consists of 1604 individuals, a subset of the 5,065 individuals who underwent CMR examination as part of the pilot phase (April 2014 –August 2015) of the UK Biobank imaging enhancement. All participants provided written consent; UK Biobank’s scientific protocol and operational procedures were reviewed and approved by the North West Multi-centre Research Ethics Committee in the UK [14]. The research presented here was conducted under access application 2964 and was approved by the UK Biobank access committee.

### Exclusion criteria

Male participants (n = 2356), female participants not reporting having undergone menopause (n = 693), participants reporting myocardial infarction, angina, heart failure, arrhythmias (including atrial fibrillation), cardiomyopathy, stroke or peripheral vascular disease (n = 76), and participants using MHT for < 3 years or with missing duration data (n = 246) were excluded from the analysis leaving a study population of 1604.

### CMR protocol and image analysis

The UK Biobank CMR protocol has been described in detail elsewhere [16]. Briefly, all examinations were performed on a wide-bore 1.5 Tesla scanner (MAGNETOM Aera, Syngo Platform VD13A, Siemens Healthcare, Erlangen, Germany). For cardiac function, long axis cines and a complete short axis stack of balanced steady-state free precession (bSSFP) cines, covering the left and right ventricle were acquired.

Analysis of the cardiac chambers for all CMR examinations was performed manually across two core laboratories according to pre-approved standard operating procedures using cvi^42^ post-processing software (Version 5.1.1, Circle Cardiovascular Imaging Inc., Calgary, Canada) by observers blinded to all exposures. LV papillary muscles were included in blood pool volumes and excluded from LV mass. Detailed descriptions of analysis methodology, including reference ranges, exemplar contours and intra- and inter-observer variability, have been previously described [17]. The CMR parameters examined in this study were left ventricular end-diastolic volume, end-systolic volume, stroke volume, ejection fraction and mass and left atrial maximal volume.

### Hormone replacement therapy and menopause

To reliably assess the impact of MHT on cardiac structure and function, only women using MHT for ≥ 3 years were included in the analysis. Data concerning MHT use was derived from UK Biobank fields 3546 (age last used MHT) and 3536 (age started MHT). Duration of MHT use was calculated by subtracting values in these two fields. Where women indicated that they were still currently using MHT, age at the time of imaging visit was used to determine duration. Duration of menopause was calculated by subtracting age at menopause (data field 3581) from age at time of imaging visit. To assess the impact of the “timing hypothesis”, a timing variable was created defined as age of menopause subtracted from age started MHT expressed in years.

### Statistical analyses

Descriptive statistics for continuous variables were presented as mean ± standard deviation or median and interquartile range (IQR) whilst categorical variables were presented as number (percentage). Differences in means were tested using unpaired t-test or Mann-Whitney-U test and differences in percentages using chi-squared test. CMR parameters used as dependent variables were LV end-diastolic volume, LV end-systolic volume, LV stroke volume, LV ejection fraction, LV mass, and LA maximal volume. All dependent variables were assessed for normality using histograms and quantile-quantile plots; natural logarithmic transformation was performed for all dependent variables barring LV ejection fraction. For each dependent variable, outliers were defined as measurements more than three interquartile ranges below the first quartile or above the third quartile and removed from analysis. With respect to missing values, the data presented is a complete case analysis.

To examine the impact of MHT use on cardiac structure and function, multivariable linear regression models were fitted for each cardiac (dependent) variable. With our sample size, the study has 80% power at the 5% significance level to detect a 0.15 standard deviation difference in any of the continuous variables; this would be considered a small effect size [18]. Co-variates included in the model (Model 1) were age, age at menopause, ethnicity, height, weight, systolic blood pressure, diastolic blood pressure, smoking status, regular alcohol use, presence of raised cholesterol, presence of diabetes, Townsend deprivation index and income. Height and weight were included as covariates in the model rather than indexing the dependent variables, as the use of ratios in regression analysis can lead to spurious results and misinterpretation [19,20]. The adjustment made ensured all variables in the model were appropriately adjusted for body composition. The variance inflation factor was calculated to test for multicollinearity. Where cardiac variables had been log-transformed, the beta coefficients were anti-logged and expressed as a percentage difference.

To determine whether the effect of MHT use on each of the cardiac outcomes differed by age, multivariable regression models were constructed with a cross-product of age and MHT fitted as an interaction term. Co-variates included: duration of MHT use fitted as thirds, ethnicity, height, weight, systolic blood pressure, diastolic blood pressure, smoking status, regular alcohol use, presence of raised cholesterol, presence of diabetes, Townsend deprivation index and income (Model 2). Interactions were tested using age as a continuous variable. For ease of interpretation we also fitted the interaction using tertile of age and presented effect sizes for MHT use by tertile.

The effect of multiple testing was considered by determining false discovery rates using the Benjamini–Yekutieli procedure in order to establish the proportion of the rejected hypotheses that are likely to be true positives.

In sensitivity analyses, to examine differences between the MHT use ≥ 3 years and no MHT use groups on CMR parameters, propensity-score matching was used. Matching was performed using all co-variates used in Model 1 at a one-to-one ratio. Differences between MHT use ≥ 3 years and propensity-matched controls was assessed using paired t-test.

To examine the effect of missing data, multiple imputation by chained equations was used to impute 20 complete datasets on which the analysis was repeated and the results pooled. Predictive mean matching with five nearest neighbours was used for continuous variables and logistic regression for binary variables. Plots were examined to assess convergence and plausibility of estimates.

All statistical analyses were performed using R (version 3.3.2) [21].

## Results

**Fig 1:**
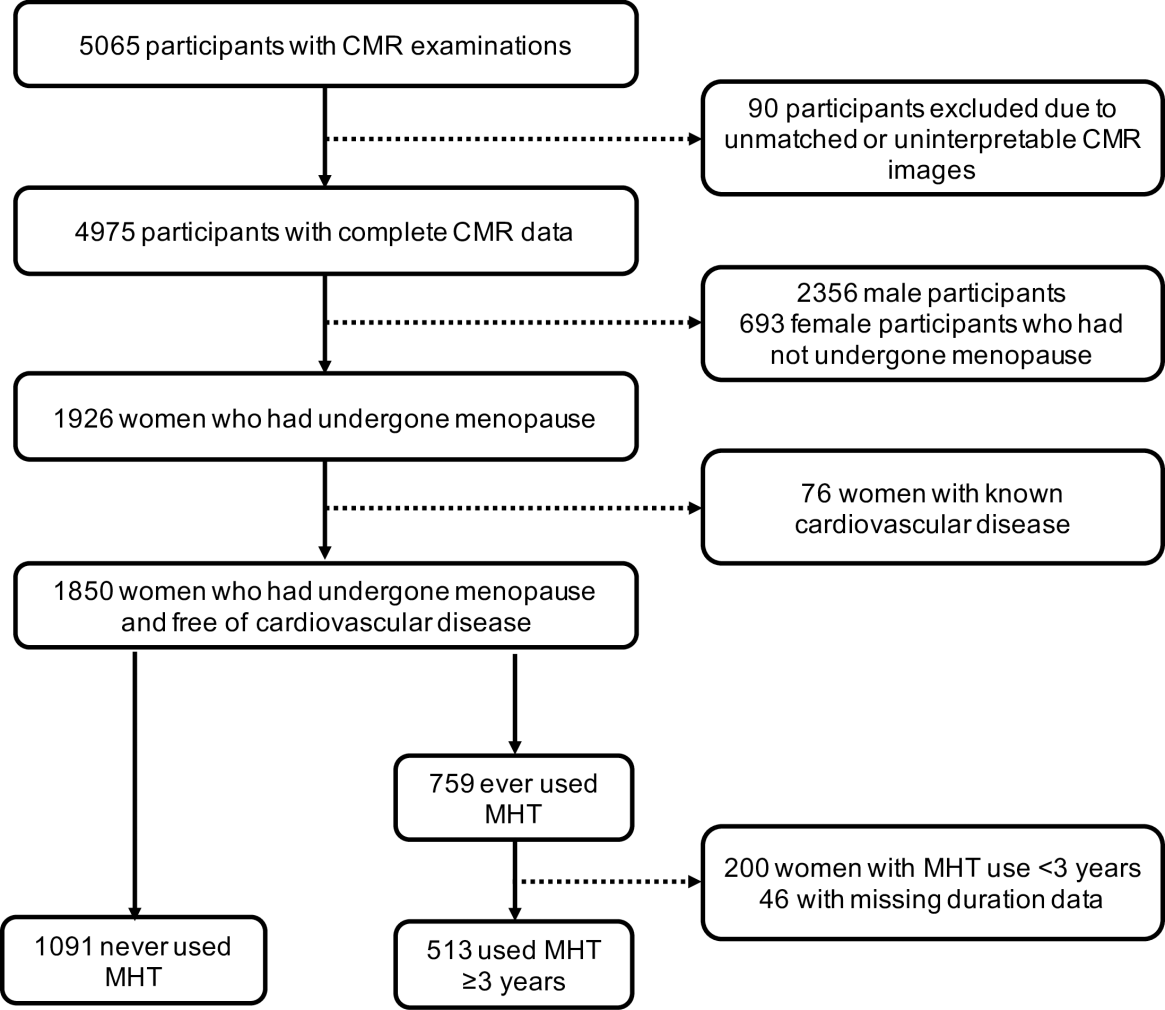
Case selection flowchart.

A total of 1,604 participants were included in this study, 513 post-menopausal women who had used MHT for ≥ 3 years and 1,091 post-menopausal women who had never used MHT; case selection is depicted in Fig 1. The mean number of outliers for CMR variables was 2 (range = 0–7).

Baseline characteristics for the study population, divided by never used MHT vs MHT use ≥ 3 years, are presented in Table 1. The mean age (65.4±5.7 vs 61.3±6.4; P<0.0001) was higher and the median age at menopause (50 [IQR = 45–52] vs 51 [IQR = 48–53]; p<0.0001) was lower in the MHT cohort compared to the never used MHT cohort. For those using MHT, the median number of years of MHT use was 8 (IQR = 5–11) and the mean age of commencement was 47.6±5.3. At the time of CMR examination, 15.2% (n = 78) were still on treatment. There was no significant difference in socioeconomic status measures between MHT users and non-users including Townsend deprivation index, household income or educational attainment.

**Table 1:** Baseline characteristics.

	Never used MHT	MHT use ≥ 3 years
	(n = 1091)	(n = 513)
**Age, years**	61.3 (6.4)	65.4 (5.7)
**BMI, kg/m**^**2**^	26.0 (4.7)	25.9 (4.3)
**Ethnicity (% Caucasian)**	92% (1004)	93% (475)
**Height, cm**	163 (6.2)	162 (6.1)
**SBP, mmHg**	133 (18.8)	135 (19.3)
**DBP, mmHg**	77 (9.9)	76 (10.0)
**Hypercholesterolaemia**	11.9% (130)	19.5% (100)
**Diabetes**	3.7% (40)	4.1% (21)
**Current smoker**	2.8% (31)	3.7% (19)
**Regular alcohol**	16.6% (154)	22.9% (103)
**Mean age at menopause (years)**	50.6 (4.3)	48.3 (6.4)
**Median age at menopause (years)**	51 [48–53]	50 [45–52]
**Mean age commenced MHT (years)**	N/A	47.6 (5.3)
**Median duration of MHT (years)**	N/A	8 [5–11]

*Indicates data presented as median (interquartile range)

Mean CMR parameters for each cohort are presented in Table 2. Before adjustment, women who had used MHT ≥ 3 years had significantly smaller LV end-diastolic volume (117±22 ml vs 124±22 ml; p<0.0001), LV end-systolic volume (46±12 ml vs 48±12 ml; p <0.005), LV stroke volume (71±14 ml vs 75±14 ml; p<0.0001) and LA maximal volume (57±17 ml vs 61±17 ml; p<0.0001) compared to the cohort who had never used MHT. There was no significant difference in LV mass between the two groups.

**Table 2:** Mean CMR parameters, unadjusted.

	Never used MHT	MHT use ≥ 3 years	P value
	(n = 1091)	(n = 513)	
**LV end-diastolic volume**	123.7 (21.4)	117.6 (21.6)	<0.0001
**LV end-systolic volume**	47.9 (12.0)	45.8 (11.9)	0.002
**LV stroke volume**	74.9 (13.6)	70.7 (14.2)	<0.0001
**LV ejection fraction**	60.9 (5.7)	60.5 (6.2)	0.313
**LV mass**	72.1 (13.7)	71.3 (14.1)	0.325
**LA maximal volume**	60.9 (17.2)	56.9 (16.8)	<0.0001

The effect of MHT use on LV and LA CMR parameters in fully adjusted models is detailed in Table 3. Use of MHT for ≥ 3 years was associated with a significant reduction in LV end-diastolic volume (123 ml vs 120 ml, effect size = -2.4%, 95% confidence interval [CI]: -4.2% to -0.5%; p = 0.013), LV stroke volume (74 ml vs 72 ml, effect size = -3.1%, 95% CI: -5.1% to -1.0%; p = 0.004) and LA maximal volume (60 ml vs 58 ml, effect size = -4.5%, 95% CI: -7.8% to -1.0%; p = 0.012). These associations remained significant at a false discovery rate of 10%. To further examine these associations produced by complete case analysis, multiple imputation of missing values was performed and the analysis repeated. The same CMR variables demonstrated significant associations with MHT use; these results are detailed in S1 Table.

**Table 3:** Effect of MHT use ≥ 3 years on CMR parameters in fully-adjusted models.

	Adjusted mean: never used MHT	Adjusted mean: MHT use > = 3 years	Effect size (%)	95% Confidence Interval	p value
**LV end-diastolic volume**	122.8	119.8	-2.4	(-4.2, -0.5)	0.013
**LV end-systolic volume**	47.5	46.9	-1.3	(-4.2,1.6)	0.383
**LV stroke volume**	74.3	72.1	-3.1	(-5.1, -1.0)	0.004
**LV ejection fraction**	60.9	60.4	-0.5	(-1.2, 0.3)	0.212
**LV mass**	71.5	71.6	0.1	(-1.9, 2.2)	0.914
**LA maximal volume**	60.2	57.5	-4.5	(-7.8, -1.0)	0.012

All parameters barring LV ejection fraction have been log-transformed and are therefore expressed as percentage change.

Model adjusted for age, age at menopause, ethnicity, height, weight, systolic blood pressure, diastolic blood pressure, smoking status, regular alcohol use, presence of raised cholesterol, presence of diabetes, Townsend deprivation index and income.

**Fig 2:**
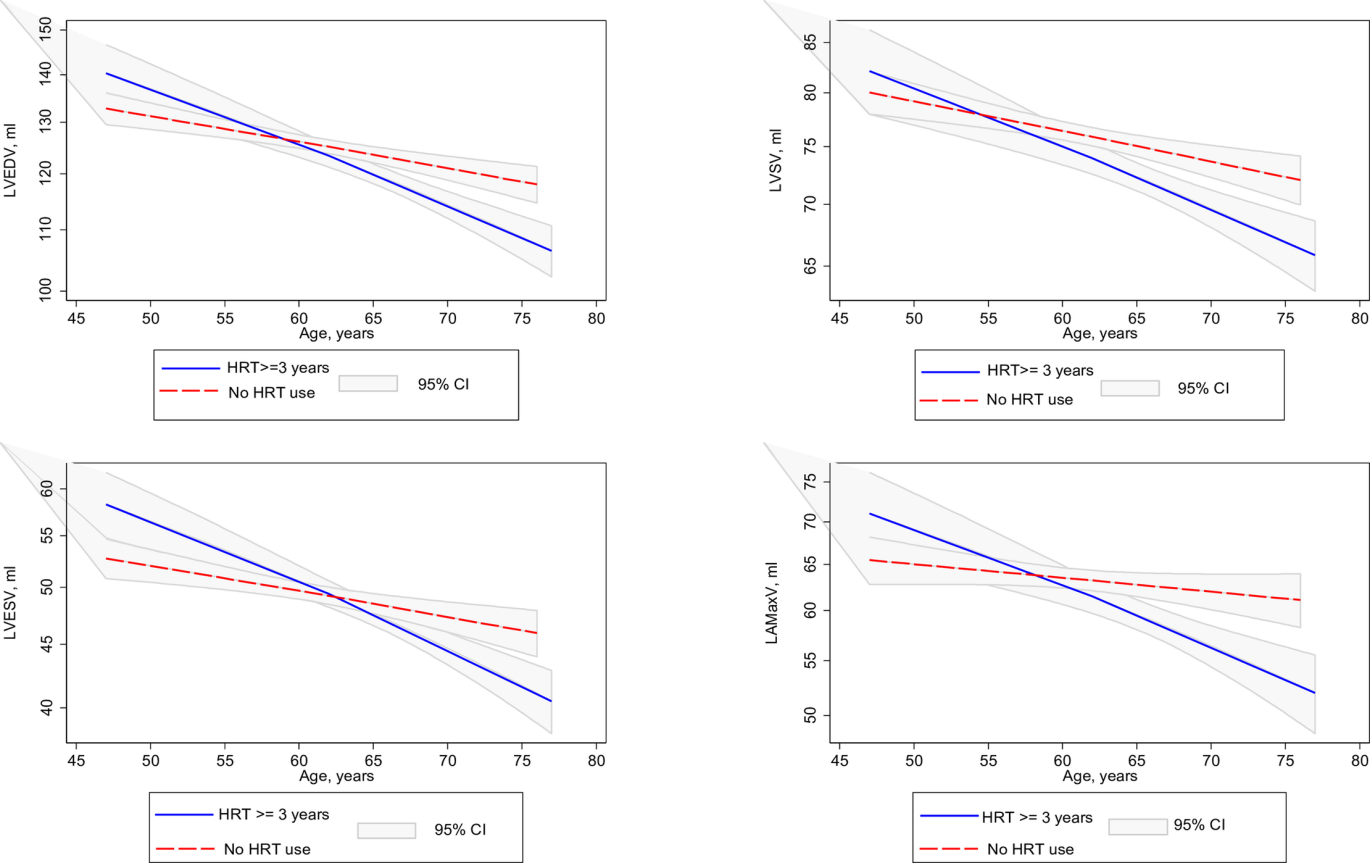
Interaction plots for age and MHT use. For every ten-year increment in age, there is a reduction in LV end-diastolic volume, LV end-systolic volume, LV stroke volume and LA maximal volume. The relationship between age and CMR outcomes is of greater magnitude amongst MHT users than that amongst non-users.

Results of interaction analyses using age*MHT as an interaction term in our regression models are presented in Table 4 and Fig 2. Age significantly modified the association between MHT use and CMR parameters with smaller chambers observed with advancing tertiles of age: LV end-diastolic volume (47–60 years: β = 3.2%, 95% CI: -0.6% to 7.2%; 61–66 years: β = -2.9%, 95% CI: -5.8% to 0.2%; 67–77 years: β = -6.2%, 95% CI: -9.0% to -3.3%; p for interaction = 0.0005), LV end-systolic volume (47–60 years: β = 7.7%, 95% CI: 1.6% to 14.1%; 61–66 years: β = -3.1%, 95% CI: —7.6% to 1.6%; 67–77 years: β = -5.9%, 95% CI: -10.1% to -1.4%; p for interaction = 0.001), LV stroke volume (47–60 years: β = 0.3%, 95% CI: -3.8% to 4.5%; 61–66 years: β = -2.6%, 95% CI: -5.9% to 0.8%; 67–77 years: β = -6.4%, 95% CI: -9.4% to -3.2%; p for interaction = 0.033), and LA maximal volume (47–60 years: β = 0.1%, 95% CI: -6.6% to 7.3%; 61–66 years: β = -1.2%, 95% CI: -6.6% to 4.6%; 67–77 years: β = -10.1%, 95% CI: -15.0% to -5.0%; p for interaction = 0.006).

**Table 4:** Effect modification analysis; interaction of MHT use with age.

	Tertile of age	MHT effect size	95% Confidence Interval	P value	P value for interaction with age
**LV end-diastolic volume**	1) 47–60 years	3.2%	(-0.6, 7.2)	0.096	p = 0.0005
	2) 61–66 years	-2.9%	(-5.8, 0.2)	0.064	
	3) 67–77 years	-6.2%	(-9.0, -3.3)	<0.0001	
**LV end-systolic volume**	1) 47–60 years	7.7%	(1.6, 14.1)	0.012	p = 0.001
	2) 61–66 years	-3.1%	(-7.6, 1.6)	0.195	
	3) 67–77 years	-5.9%	(-10.1, -1.4)	0.010	
**LV stroke volume**	1) 47–60 years	0.3%	(-3.8, 4.5)	0.896	p = 0.033
	2) 61–66 years	-2.6%	(-5.9, 0.8)	0.128	
	3) 67–77 years	-6.4%	(-9.4, -3.2)	<0.0001	
**LA maximal volume**	1) 47–60 years	0.1%	(-6.6, 7.3)	0.974	p = 0.006
	2) 61–66 years	-1.2%	(-6.6, 4.6)	0.688	
	3) 67–77 years	-10.1%	(-15.0, -5.0)	0.0002	

Effect sizes presented following adjustment for: duration of MHT use fitted as tertiles, age at menopause, ethnicity, height, weight, systolic blood pressure, diastolic blood pressure, smoking status, regular alcohol use, presence of raised cholesterol, presence of diabetes, Townsend score and income.

Results from sensitivity analyses examining the difference between CMR parameters in MHT users ≥ 3 years and propensity-matched controls are presented in Table 5. There were 429 matched participants in each group. As in multivariable regression models (Table 3), LV end-diastolic volume (MHT users = 117.5 ml vs never users = 121.7 ml, mean difference = -3.2%, 95% CI: -5.5% to -0.9%; p = 0.007), LV stroke volume (MHT users = 70.9 ml vs never users = 73.8 ml, mean difference = -3.7%, 95% CI: -6.1% to -1.3%; p = 0.003) and LA maximal volume (MHT users = 56.8 ml vs never users = 59.4 ml, mean difference = -4.9%, 95% CI: -8.9% to -0.8%; p = 0.019) were significantly lower in MHT users ≥3 years. S2 Table details the balance between the co-variates both before and after propensity-matching.

**Table 5:** Sensitivity analysis: Mean difference in CMR parameters by MHT users ≥ 3 years and propensity-matched controls.

	Mean (SD) in MHT users ≥3 years (n = 429)	Mean (SD) in propensity-matched controls (n = 429)	Mean difference (%)	95% confidence interval	P value
**LV end-diastolic volume**	117.5 (21.6)	121.7 (19.9)	-3.2	(-5.5, -0.9)	0.007
**LV end-systolic volume**	45.6 (11.8)	47.0 (11.9)	-2.5	(-5.8, 1.0)	0.157
**LV stroke volume**	70.9 (14.2)	73.8 (12.7)	-3.7	(-6.1, -1.3)	0.003
**LV ejection fraction**	60.8 (6.1)	61.0 (6.1)	-0.4	(-1.2, 0.5)	0.394
**LV mass**	70.9 (14.2)	71.6 (13.2)	-0.7	(-3.2, 2.0)	0.601
**LA maximal volume**	56.8 (17.0)	59.4 (17.3)	-4.9	(-8.9, -0.8)	0.019

Propensity-matched controls are selected using the following variables: age, age at menopause, ethnicity, height, body mass index, systolic blood pressure, diastolic blood pressure, smoking status, regular alcohol use, presence of raised cholesterol and presence of diabetes, Townsend deprivation index and income.

## Discussion

In a population-based cohort of 1604 post-menopausal women free of known cardiovascular disease, the present study identified the following: firstly, LV end-diastolic volumes and LA maximal volumes were lower in women using MHT ≥ 3 years compared to those who had never used MHT after accounting for potential confounders. Secondly, MHT use significantly modified the relationship between advancing age and LV end-diastolic volume, LV end-systolic volume and LA maximal volume. Thirdly, timing of commencement of MHT in relation to the onset of menopause had no discernible impact of LV or LA volumes.

This study describes the relationship between MHT use and prognostically important cardiac phenotypes and indicates lower LV end-diastolic and LA maximal volumes in women who used MHT ≥ 3 years compared to those who never received treatment. Increases in LV volumes–LV dilatation–is associated with cardiac decompensation and poor prognosis in a range of cardiovascular diseases. Increases in end-diastolic volume and end-systolic volume have been demonstrated to be associated with an increased risk of heart failure in asymptomatic individuals [22] whilst reduction in LV volumes–indicative of reverse cardiac remodelling–is associated with favourable outcomes [23,24]. Equivalently, LA enlargement, as determined by LA maximal volume, is a robust and independent predictor of incident cardiovascular events [25,26] whilst reduction in LA volumes (reverse remodelling) is associated with lower mortality and risk of heart failure [27]. There was no significant difference in LV mass between MHT users ≥ 3 years and those who had never used MHT. LV mass is one of the most important cardiovascular imaging-derived phenotypes with increases in LV mass predicting a higher incidence of cardiovascular events and mortality [28].

This study demonstrates that use of MHT significantly impacts upon age-related reduction in LV and LA volumes. Recently published data detailing reference ranges for CMR imaging derived from a strictly healthy UK Biobank cohort has demonstrated that LV end-diastolic volume, end-systolic volume and LA maximal volume all decrease with advancing age [17], in keeping with findings from the never used MHT cohort. It is noteworthy, however, that use of MHT results in a much more marked rate of diminishment in LV and LA volumes, even after accounting for duration of MHT in addition to other confounders. Given that only 15% of women were using MHT at the time of CMR examination, it appears that this is an effect that persists, rather than being a contemporaneous result of being on treatment.

CMR is the most accurate and reproducible cardiac imaging modality and, when coupled with a large cohort size, permits detection of subclinical changes in cardiac structure and function. Whilst these subtle alterations may not result in any discernible impact to an individual at a single point in time, if they persist it is possible that they will eventually lead to prognostically relevant changes in cardiovascular outcomes. To our knowledge, this study is the first to explore the relationship between MHT use and subclinical changes in cardiac structure and function. Previous studies examining the impact of MHT on the cardiovascular system at the subclinical stage have focused on atherosclerotic burden as detected by cardiac computed tomography (CT). The most influential of these was a substudy of the Women’s Health Initiative which reported the coronary artery calcified-plaque burden was lower in women assigned to MHT than in those assigned to placebo [29]. The more recent Early versus Late Intervention Trial with Estradiol (ELITE) study [30], designed specifically to investigate the “timing hypothesis” in relation to atherosclerosis progression in post-menopausal women, did not show any difference in plaque burden as assessed by cardiac CT in either early (<6 years) or late (≥10 years) menopause when compared to placebo although the authors did state that their sample size may be insufficient to detect any difference.

Despite landmark randomised trials and systematic reviews [31] declaring that MHT does not provide any protective effects from cardiac events or mortality, this has been challenged by more recent studies examining both clinical end-points and surrogate markers of atherosclerosis progression. What is clear is that there remains significant confusion regarding MHT’s benefit, or lack thereof, in relation to cardiovascular health. Discouragingly, it has been noted that it is “unlikely” that additional large, prospective trials will be performed investigating MHT’s impact on cardiovascular disease due to existing controversy, fear of potential harm and the expense associated with longitudinal follow-up [32]. We hope that our utilisation of biomarkers provided by CMR in the context of a large-scale, population-based study such as UK Biobank has provided a useful and novel method of examining MHT’s influence on the cardiovascular system.

This study used a large population-based cohort, with uniform assessment of exposures, covariates and outcomes. Importantly, there was no difference in socioeconomic status between the two groups; previous studies have highlighted that women prescribed MHT are of higher social class and educational attainment–and therefore, in general, healthier–than women who do not receive MHT, thereby confounding results [33,34]. This is something that we have attempted to control for in this data. However, there are several limitations that should be considered in the interpretation of the findings. Firstly, the analysis was cross-sectional, thus causality cannot be inferred from the associations demonstrated. Secondly, it was not possible to explore longitudinal change in cardiac structure in relation to MHT use. Thirdly, all menopause and MHT data was self-reported. Finally, we were not able to provide data on type of MHT due to significant amount of missing data.

## Conclusions

In a large, population-based cohort of post-menopausal women free of cardiovascular disease, use of MHT is not associated with adverse, subclinical changes in cardiac structure and function. Indeed, we demonstrate significantly smaller LV and LA chamber volumes which have been linked to favourable cardiovascular outcomes in other settings. Our findings provide a novel way to examine the impact of MHT on the cardiovascular system; future work will focus upon linkage of MHT use and CMR parameters to cardiovascular outcome data.

## Supporting information

S1 Table Effect of MHT use ≥ 3 years on CMR parameters in fully-adjusted models after multiple imputation of missing values. Data was partially missing for 275/1604 (17%) of participants. Between those with missing and no missing data, the groups were similar for all co-variates other than BMI (25.8 kg/m^2^ vs. 26.8 kg/m^2^, p = 0.003) and systolic blood pressure (133 mmHg vs. 137 mmHg, p = 0.009) which were higher in those with missing data. After multiple imputation of missing values, the effect sizes are similar to those from complete case analysis with the same CMR parameters realising significance. The complete case analysis detailed in the main manuscript provides more conservative results.
(DOCX)

S2 Table Co-variate balance before and after propensity-matching. Propsensity matching was performed using “MatchIt” package in R. Optimal matching technique was used.
(DOCX)

## Abbreviations

BMI: body mass index
bSSFP: balanced steady-state free precession
CMR: cardiovascular magnetic resonance
CVD: cardiovascular disease
HERS: Heart and Estrogen/Progestin Replacement Study
LA: left atrium
LV: left ventricle
MHT: menopausal hormone therapy
WHI: Women’s Health Initiative

## References

[1] Sidney S, Petitti DB, Quesenberry CP. Myocardial infarction and the use of estrogen and estrogen-progestogen in postmenopausal women. Ann Intern Med. 1997;127: 501–8. Available: http://www.ncbi.nlm.nih.gov/pubmed/9313017 9313017 10.7326/0003-4819-127-7-199710010-00001

[2] Stampfer MJ, Willett WC, Colditz GA, Rosner B, Speizer FE, Hennekens CH. A Prospective Study of Postmenopausal Estrogen Therapy and Coronary Heart Disease. N Engl J Med. 1985;313: 1044–1049. doi: 10.1056/NEJM198510243131703 4047106 10.1056/NEJM198510243131703

[3] Stampfer MJ, Colditz GA. Estrogen replacement therapy and coronary heart disease: a quantitative assessment of the epidemiologic evidence. Prev Med (Baltim). 1991;20: 47–63. Available: http://www.ncbi.nlm.nih.gov/pubmed/1826173 10.1016/0091-7435(91)90006-p 1826173

[4] Hulley S, Grady D, Bush T, Furberg C, Herrington D, Riggs B, et al Randomized trial of estrogen plus progestin for secondary prevention of coronary heart disease in postmenopausal women. Heart and Estrogen/progestin Replacement Study (HERS) Research Group. JAMA. 1998;280: 605–13. Available: http://www.ncbi.nlm.nih.gov/pubmed/9718051 9718051 10.1001/jama.280.7.605

[5] Rossouw JE, Anderson GL, Prentice RL, LaCroix AZ, Kooperberg C, Stefanick ML, et al Risks and benefits of estrogen plus progestin in healthy postmenopausal women: principal results From the Women’s Health Initiative randomized controlled trial. JAMA. 2002;288: 321–33. Available: http://www.ncbi.nlm.nih.gov/pubmed/12117397 12117397 10.1001/jama.288.3.321

[6] Grady D, Herrington D, Bittner V, Blumenthal R, Davidson M, Hlatky M, et al Cardiovascular disease outcomes during 6.8 years of hormone therapy: Heart and Estrogen/progestin Replacement Study follow-up (HERS II). JAMA. 2002;288: 49–57. Available: http://www.ncbi.nlm.nih.gov/pubmed/12090862 12090862 10.1001/jama.288.1.49

[7] American College of Obstetricians and Gynecologists. ACOG committee opinion American College of Obstetricians and Gynecologists;

[8] Pines A. Guidelines and recommendations on hormone therapy in the menopause. J Midlife Health. 2010;1: 41 doi: 10.4103/0976-7800.66990 21799639 10.4103/0976-7800.66990 PMC3139264

[9] Schierbeck LL, Rejnmark L, Tofteng CL, Stilgren L, Eiken P, Mosekilde L, et al Effect of hormone replacement therapy on cardiovascular events in recently postmenopausal women: randomised trial. BMJ. 2012;345: e6409 Available: http://www.ncbi.nlm.nih.gov/pubmed/23048011 doi: 10.1136/bmj.e6409 23048011 10.1136/bmj.e6409

[10] Marjoribanks J, Farquhar C, Roberts H, Lethaby A. Trial does not change the conclusions of Cochrane review of long term hormone therapy for perimenopausal and postmenopausal women. BMJ. 2012;345: e8141–e8141. doi: 10.1136/bmj.e8141 23208255 10.1136/bmj.e8141

[11] Manson JE, Aragaki AK, Rossouw JE, Anderson GL, Prentice RL, LaCroix AZ, et al Menopausal Hormone Therapy and Long-term All-Cause and Cause-Specific Mortality. JAMA. American Medical Association; 2017;318: 927 doi: 10.1001/jama.2017.11217 28898378 10.1001/jama.2017.11217 PMC5728370

[12] Barrett-Connor E. Hormones and heart disease in women: the timing hypothesis. Am J Epidemiol. 2007;166: 506–10. Available: http://www.ncbi.nlm.nih.gov/pubmed/17849510 17849510 10.1093/aje/kwm214

[13] Clarkson TB, Meléndez GC, Appt SE. Timing hypothesis for postmenopausal hormone therapy. Menopause J North Am Menopause Soc. 2013;20: 342–353. doi: 10.1097/GME.0b013e3182843aad 23435033 10.1097/GME.0b013e3182843aad

[14] Sudlow C, Gallacher J, Allen N, Beral V, Burton P, Danesh J, et al UK biobank: an open access resource for identifying the causes of a wide range of complex diseases of middle and old age. PLoS Med. Public Library of Science; 2015;12: e1001779 doi: 10.1371/journal.pmed.1001779 25826379 10.1371/journal.pmed.1001779 PMC4380465

[15] Petersen SE, Matthews PM, Bamberg F, Bluemke DA, Francis JM, Friedrich MG, et al Imaging in population science: cardiovascular magnetic resonance in 100,000 participants of UK Biobank—rationale, challenges and approaches. J Cardiovasc Magn Reson. 2013;15: 46 doi: 10.1186/1532-429X-15-46 23714095 10.1186/1532-429X-15-46 PMC3668194

[16] Petersen SE, Matthews PM, Francis JM, Robson MD, Zemrak F, Boubertakh R, et al UK Biobank’s cardiovascular magnetic resonance protocol. J Cardiovasc Magn Reson. BioMed Central Ltd; 2016;18: 8 doi: 10.1186/s12968-016-0227-4 26830817 10.1186/s12968-016-0227-4 PMC4736703

[17] Petersen SE, Aung N, Sanghvi MM, Zemrak F, Fung K, Paiva JM, et al Reference ranges for cardiac structure and function using cardiovascular magnetic resonance (CMR) in Caucasians from the UK Biobank population cohort. J Cardiovasc Magn Reson. BioMed Central; 2017;19: 18 doi: 10.1186/s12968-017-0327-9 28178995 10.1186/s12968-017-0327-9 PMC5304550

[18] Cohen J. Statistical Power Analysis for the Behavioral Sciences [Internet]. Behavioral Sciences, Lawrence Erlbaum Associates Inc., Hillsdale, NJ Academic Press; 1988 doi: 10.2307/2286629

[19] Kronmal RA. Spurious Correlation and the Fallacy of the Ratio Standard Revisited. J R Stat Soc Ser A (Statistics Soc. WileyRoyal Statistical Society; 1993;156: 379 doi: 10.2307/2983064

[20] Tu Y-K, Clerehugh V, Gilthorpe MS. Ratio variables in regression analysis can give rise to spurious results: illustration from two studies in periodontology. J Dent. 2004;32: 143–51. Available: http://www.ncbi.nlm.nih.gov/pubmed/14749086 14749086 10.1016/j.jdent.2003.09.004

[21] R Core Team. R: A Language and Environment for Statistical Computing [Internet]. Vienna, Austria: R Foundation for Statistical Computing; 2017 Available: https://www.r-project.org

[22] Vasan RS, Larson MG, Benjamin EJ, Evans JC, Levy D. Left Ventricular Dilatation and the Risk of Congestive Heart Failure in People without Myocardial Infarction. N Engl J Med. Massachusetts Medical Society; 1997;336: 1350–1355. doi: 10.1056/NEJM199705083361903 9134875 10.1056/NEJM199705083361903

[23] Cohn JN, Ferrari R, Sharpe N. Cardiac remodeling—concepts and clinical implications: a consensus paper from an international forum on cardiac remodeling. J Am Coll Cardiol. 2000;35: 569–582. doi: 10.1016/S0735-1097(99)00630-0 10716457 10.1016/s0735-1097(99)00630-0

[24] Kerkhof PL. Characterizing heart failure in the ventricular volume domain. Clin Med Insights Cardiol. Libertas Academica; 2015;9: 11–31. doi: 10.4137/CMC.S18744 25780344 10.4137/CMC.S18744 PMC4345934

[25] Kizer JR, Bella JN, Palmieri V, Liu JE, Best LG, Lee ET, et al Left atrial diameter as an independent predictor of first clinical cardiovascular events in middle-aged and elderly adults: The Strong Heart Study (SHS). Am Heart J. 2006;151: 412–418. doi: 10.1016/j.ahj.2005.04.031 16442908 10.1016/j.ahj.2005.04.031

[26] Tsang TSM, Abhayaratna WP, Barnes ME, Miyasaka Y, Gersh BJ, Bailey KR, et al Prediction of Cardiovascular Outcomes With Left Atrial Size. J Am Coll Cardiol. 2006;47: 1018–1023. doi: 10.1016/j.jacc.2005.08.077 16516087 10.1016/j.jacc.2005.08.077

[27] Mathias A, Moss AJ, McNitt S, Zareba W, Goldenberg I, Solomon SD, et al Clinical Implications of Complete Left-Sided Reverse Remodeling With Cardiac Resynchronization Therapy. J Am Coll Cardiol. 2016;68: 1268–1276. doi: 10.1016/j.jacc.2016.06.051 27634117 10.1016/j.jacc.2016.06.051

[28] Levy D, Garrison RJ, Savage DD, Kannel WB, Castelli WP. Prognostic implications of echocardiographically determined left ventricular mass in the Framingham Heart Study. N Engl J Med. 1990;322: 1561–6. doi: 10.1056/NEJM199005313222203 2139921 10.1056/NEJM199005313222203

[29] Manson JE, Allison MA, Rossouw JE, Carr JJ, Langer RD, Hsia J, et al Estrogen Therapy and Coronary-Artery Calcification. N Engl J Med. Massachusetts Medical Society; 2007;356: 2591–2602. doi: 10.1056/NEJMoa071513 17582069 10.1056/NEJMoa071513

[30] Hodis HN, Mack WJ, Henderson VW, Shoupe D, Budoff MJ, Hwang-Levine J, et al Vascular Effects of Early versus Late Postmenopausal Treatment with Estradiol. N Engl J Med. Massachusetts Medical Society; 2016;374: 1221–1231. doi: 10.1056/NEJMoa1505241 27028912 10.1056/NEJMoa1505241 PMC4921205

[31] Boardman HM, Hartley L, Eisinga A, Main C, Roqué i Figuls M, Bonfill Cosp X, et al Hormone therapy for preventing cardiovascular disease in post-menopausal women In: Boardman HM, editor. Cochrane Database of Systematic Reviews. Chichester, UK: John Wiley & Sons, Ltd; 2015 p. CD002229 doi: 10.1002/14651858.CD002229.pub4 10.1002/14651858.CD002229.pub4 PMC10183715 25754617

[32] Harvey RE, Coffman KE, Miller VM. Women-specific factors to consider in risk, diagnosis and treatment of cardiovascular disease. Womens Health (Lond Engl). NIH Public Access; 2015;11: 239–57. doi: 10.2217/whe.14.64 25776297 10.2217/whe.14.64 PMC4386625

[33] Posthuma WF, Westendorp RG, Vandenbroucke JP. Cardioprotective effect of hormone replacement therapy in postmenopausal women: is the evidence biased? BMJ. BMJ Publishing Group; 1994;308: 1268–9. Available: http://www.ncbi.nlm.nih.gov/pubmed/8205018 8205018 10.1136/bmj.308.6939.1268 PMC2540219

[34] Rödström K, Bengtsson C, Lissner L, Björkelund C. Pre-existing risk factor profiles in users and non-users of hormone replacement therapy: prospective cohort study in Gothenburg, Sweden. BMJ. 1999;319 Available: http://www.bmj.com/content/319/7214/890.1 10.1136/bmj.319.7214.890 PMC28245 10506047

