# Supplementary material for: The impact of menopausal hormone therapy (MHT) on cardiac structure and function: Insights from the UK Biobank imaging enhancement study
Source: PLoS One. 2018 Mar 8;13(3):e0194015. doi: 10.1371/journal.pone.0194015 (PMC5843282; doi:10.1371/journal.pone.0194015)
Supplement: S1 Table — Data was partially missing for 275/1604 (17%) of participants. Between those with missing and no missing data, the groups were similar for all co-variates other than BMI (25.8 kg/m2 vs. 26.8 kg/m2, p = 0.003) and systolic blood pressure (133 mmHg vs. 137 mmHg, p = 0.009) which were higher in those with missing data. After multiple imputation of missing values, the effect sizes are similar to those from complete case analysis with the same CMR parameters realising significance. The complete case analysis detailed in the main manuscript provides more conservative results. (DOCX) [file pone.0194015.s001.docx]

**Supplementary Table 1: Effect of MHT use ≥ 3 years on CMR parameters in fully-adjusted models after multiple imputation of missing values.**

|  | **Effect size (%)** | **95% Confidence Interval** | **p value** |
| --- | --- | --- | --- |
| **LV end-diastolic volume** | -2.1 | (-3.8, -0.4) | 0.016 |
| **LV end-systolic volume** | -0.6 | (-3.4, 2.3) | 0.693 |
| **LV stroke volume** | -3.3 | (-5.1, -1.5) | 0.0004 |
| **LV ejection fraction** | -0.7 | (-1.4, 0.02) | 0.055 |
| **LV mass** | 0.6 | (-1.4, 2.5) | 0.563 |
| **LA maximal volume** | -4.5 | (-7.9, -1.5) | 0.005 |

Data was partially missing for 275/1604 (17%) of participants. Between those with missing and no missing data, the groups were similar for all co-variates other than BMI (25.8 kg/m^2^ vs. 26.8 kg/m^2^, p=0.003) and systolic blood pressure (133 mmHg vs. 137 mmHg, p=0.009) which were higher in those with missing data. After multiple imputation of missing values, the effect sizes are similar to those from complete case analysis with the same CMR parameters realising significance. The complete case analysis detailed in the main manuscript provides more conservative results.
