# Supplementary material for: The impact of menopausal hormone therapy (MHT) on cardiac structure and function: Insights from the UK Biobank imaging enhancement study
Source: PLoS One. 2018 Mar 8;13(3):e0194015. doi: 10.1371/journal.pone.0194015 (PMC5843282; doi:10.1371/journal.pone.0194015)
Supplement: S2 Table — Propsensity matching was performed using “MatchIt” package in R. Optimal matching technique was used. (DOCX) [file pone.0194015.s002.docx]

*Propsensity matching was performed using “MatchIt” package in R. Optimal matching technique was used.*


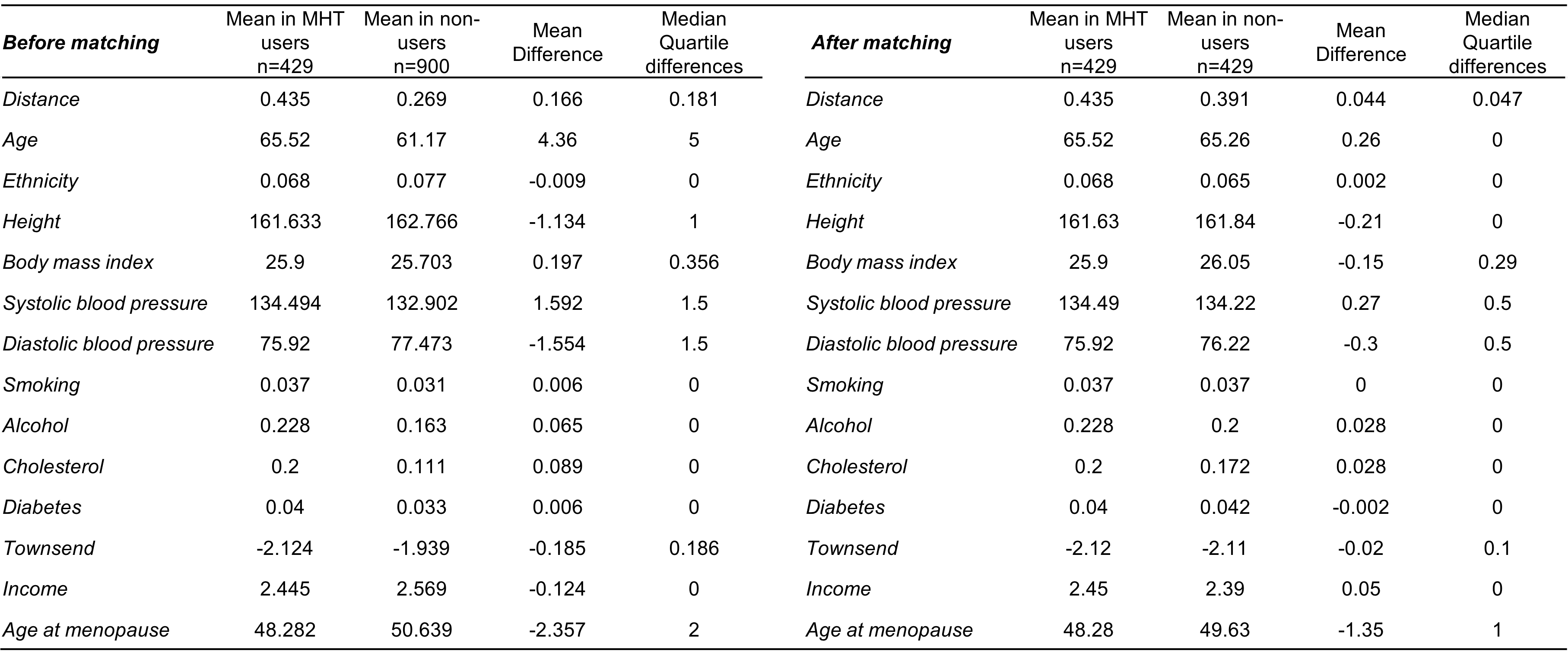


Supplementary Table 2: Co-variate balance before and after propensity-matching.
